# First-in-human phase I dose-escalation and dose-expansion trial of the selective MEK inhibitor HL-085 in patients with advanced melanoma harboring *NRAS* mutations

**DOI:** 10.1186/s12916-022-02669-7

**Published:** 2023-01-04

**Authors:** Xuan Wang, Zhiguo Luo, Jing Chen, Yu Chen, Dongmei Ji, Li Fan, Ling Chen, Qian Zhao, Pei Hu, Peng Sun, Zhongwei Jia, Jun Guo, Lu Si

**Affiliations:** 1 Key Laboratory of Carcinogenesis and Translational Research (Ministry of Education), Department of Renal Cancer and Melanoma, Peking University Cancer Hospital and Research Institute, Beijing, China; 2 Fudan University Shanghai Cancer Center, Shanghai, China; 3 Union Hospital, Tongji Medical College, Huazhong University of Science and Technology, Wuhan, China; 4 Fujian Medical University Cancer Hospital, Fuzhou, China; 5 Clinical Pharmacology Research Center, Peking Union Medical College Hospital, Chinese Academy of Medical Sciences & Peking Union Medical College, State Key Laboratory of Complex Severe and Rare Diseases, NMPA Key Laboratory for Clinical Research and Evaluation of Drug, Beijing Key Laboratory of Clinical PK and PD Investigation for Innovative Drugs, Beijing, China; 6 Department of Clinical Research and Development, Shanghai Kechow Pharma, Inc., Shanghai, China

**Keywords:** HL-085, MEK inhibitor, Advanced melanoma, *NRAS* mutation, Circulating tumor DNA

## Abstract

**Background:**

HL-085 is a selective, orally administered MEK1/2 inhibitor. We aimed to evaluate the safety and efficacy of HL-085 in patients with advanced melanoma harboring *NRAS* mutations.

**Methods:**

This was a multicenter phase 1 study. HL-085 was administered twice daily in a standard 3 + 3 dose-escalation design (10 dose cohorts; 0.5–18 mg twice daily), followed by dose expansion at the recommended phase II dose (RP2D). The primary endpoints included tolerability, dose-limiting toxicity (DLT), maximum tolerated dose (MTD) and RP2D.

**Results:**

Between September 13, 2017, and January 18, 2021, 42 patients were enrolled (dose escalation phase: *n* = 30; dose expansion phase: *n* = 12). No DLT was reported during dose escalation and MTD was not reached with HL-085 doses up to 18 mg twice daily. The RP2D was 12 mg twice daily. The most common all-grade drug-related adverse events (AEs) across all dose levels were rash (61.9%), increased creatine phosphokinase (CK, 59.5%), face edema (50.0%), increased aspartate aminotransferase (47.6%), peripheral edema (40.5%), diarrhea (33.3%), alanine aminotransferase (33.3%), and paronychia (19.0%), most of which were grade 1 and 2. Most frequency of grade ≥ 3 AEs were CK (14.2%), asthenia (7.1%), peripheral edema (4.8%), and acneiform dermatitis (4.8%). In the cohort of 12 mg twice daily dose (15 patients), confirmed objective response rate was 26.7%; disease control rate was 86.7%; median duration of response was 2.9 months; median progression-free survival was 3.6 months.

**Conclusions:**

The HL-085 showed acceptable tolerability and substantial clinical activity in patients with advanced melanoma harboring *NRAS* mutations.

**Trial registration:**

Trial registration ClinicalTrials.gov number: NCT03973151.

**Supplementary Information:**

The online version contains supplementary material available at 10.1186/s12916-022-02669-7.

## Background

Melanoma was recognized as the most dangerous type of skin cancer, especially for metastatic melanoma. Before 2011, metastatic melanoma was considered a devastating disease, the standard-of-care treatments during this time included dacarbazine chemotherapy and immunotherapy with the cytokine IL-2, and the median overall survival was only 9 months. Fortunately, the treatment landscape has shifted dramatically during recent years [1]; targeted therapies and immunotherapies have been shown to improve clinical outcomes for patients with unresectable or metastatic disease [1–5].

After *BRAF* mutations, *NRAS* mutations are the second most common oncogenic driver genetic mutations in melanoma [6–8]. BRAF inhibitors, including vemurafenib and dabrafenib, have demonstrated efficacy in patients with *BRAF*-mutated melanoma [9–11]; however, no targeted therapy has been approved for patients with melanoma harboring *NRAS* mutations to date. Since *NRAS* mutations cause aberrant activation of the mitogen-activated protein kinase pathway in melanoma [12], there is considerable interest in the development of mitogen-activated extracellular signal-related kinase (MEK) inhibitors as a novel treatment strategy that could improve the clinical outcomes of these patients. In a phase III trial, binimetinib (a selective MEK1/2 inhibitor) has shown improved progression-free survival (PFS) compared with dacarbazine in patients with *NRAS*-mutated melanoma, which is a promising treatment option for advanced melanoma; however, the main regulatory authorities have not approved the treatment, despite the positive results of the NEMO clinical trial, in view of the cost–benefit ratio [13].

HL-085 is an orally active, selective MEK inhibitor that suppresses MEK1 activity with a half-maximal inhibitory concentration (IC_50_) of 1.9–10 nM [14]. In vitro studies showed cytotoxic responses in RAS/RAF-mutated cell lines (A375, COLO-205 and HL-60) with IC_50_ less than 1 nM (unpublished investigator brochure, Shanghai KeChow Pharma.). In vivo studies demonstrated that oral administration of HL-085 at 1 mg/kg in BRAF-mutated COLO-205 and A375 xenografts models had high tumor growth inhibition value (70–76% and 60–70%, respectively) [14]. In vitro and in vivo mechanism studies showed that HL-085 inhibited kinase activity of MEK1 and prevented RAS/RAF-dependent ERK phosphorylation, hence inhibiting the growth and proliferation of tumor cells (unpublished investigator brochure, Shanghai KeChow Pharma.).

This phase I trial was first-in-human trial to evaluate the tolerability, preliminary efficacy, pharmacokinetics of HL-085 in patients with advanced *NRAS*-mutated melanoma and explored potential biomarkers of treatment efficacy. We report here the results of a phase 1 trial of HL-085.

## Methods

### Study design

This was an open-label, single-arm, dose-escalation/dose-expansion, phase I study comprising two phases: a dose-escalation phase and an expansion phase. The dose-escalation phase adopted a standard 3 + 3 design to determine the maximum tolerated dose (MTD), dose-limiting toxicity (DLT), and the recommended phase II dose (RP2D) of HL-085. In the dose-expansion phase, patients were administered at the RP2D to further evaluate the safety and efficacy of the HL-085.

This study was approved by local institutional review boards and ethics committee at each trial center and was conducted in line with good clinical practice guidelines, Declaration of Helsinki, and relevant regulations. Prior to study initiation, all patients provided written informed consent. The study protocol was registered at clinicaltrials.gov (NCT03973151).

### Patient eligibility

Eligible patients were aged 18–70 years; histologically or cytologically confirmed, unresectable, stage III or IV melanoma; with *NRAS* mutations in tissue biopsy samples available at baseline tested by centralized laboratories; with measurable lesions per Response Evaluation Criteria in Solid Tumors (RECIST) version 1.1; Eastern Cooperative Oncology Group (ECOG) performance score 0 or 1; life expectancy of > 3 months; and adequate hematologic, renal, and hepatic function.

Patients were excluded if they had active central nervous system disease except for patients with stable brain disease for ≥ 3 months following stereotactic brain radiotherapy or surgery; inability to swallow or any small intestinal resection that would preclude adequate absorption of the study drug; uncontrolled concomitant or infectious diseases; history of retinal disease; prior treatment with a specific MEK inhibitor; or known allergy to the study drug or its analogs. Strong inducers or inhibitors of CYP isozyme had to be discontinued ≥ 1 week before study treatment.

### Study procedures

HL-085 (Shanghai Kechow Pharmaceutical Research & Development Co., Ltd, Shanghai, China) was administered as 0.5 mg, 2.0 mg, and 6.0 mg capsules, twice daily (BID), orally, with a cup of warm water on an empty stomach (≥ 2 ours before or after a light meal). Based on prior data on the pharmacodynamics, pharmacokinetics, and toxicity of HL-085 in vivo and in vitro, as well as the efficacy and safety of MEK inhibitors currently under development, a starting dose of 0.5 mg BID was selected for the dose-escalation phase. The dose was then escalated in the following sequence: 1 mg BID, 2 mg BID, 3 mg BID, 4 mg BID, 6 mg BID, 9 mg BID, 12 mg BID, 15 mg BID, and 18 mg BID. The study planned to enroll at least three evaluable patients in each dose group.

Patients were evaluable if they had DLT from 7-day run-in period to the end of the first 28-day treatment cycle. DLT included the following conditions: grade 4 hematologic toxicity or grade 3 thrombocytopenia with bleeding; any grade ≥ 3 non-hematologic AEs which could not recover to ≤ 2 after treatment or with interruption of HL-085 for ≥ 14 days, including nausea, vomiting, rash, diarrhea, aspartate aminotransferase (AST)/alanine aminotransferase (ALT) elevation) abnormality; any grade ≥ 3 ocular disorders and cardiac dysfunction AEs which could not recover to grade ≤ 2 after treatment or resulted in treatment interruption for ≥ 14 days due to toxicity.

### Safety assessments

Adverse events (AEs) were monitored from the time of informed consent until 30 days after the last study drug administration. Safety assessments included evaluation of vital signs, physical examination, ophthalmic examination, ECOG performance status, clinical laboratory tests (routine blood tests, blood biochemistry, blood coagulation function and standard urinalysis), 12-lead electrocardiogram, and echocardiogram. The severity of AEs was graded according to the National Cancer Institute Common Terminology Criteria for Adverse Events (version 5.0).

### Efficacy assessments

Efficacy evaluations were performed by computed tomography or magnetic resonance imaging on baseline (within 28 days before the first dose), cycle 2 day 1, and then every 2 cycles (8 weeks) from cycle 3 day 1(whether CT or MRI is selected, the evaluation methods and parameters need to be consistent in each cycle). Response was evaluated by the investigator according to RECIST, version 1.1. The efficacy analysis was conducted in patients who have had at least one tumor evaluation, and when someone meets the partial response (PR) or complete response (CR) criteria, efficacy will be confirmed again at a subsequent time point (interval of at least 4 weeks) before CR or PR is granted.

### cfDNA extraction, library construction, and targeted sequencing

Patient blood samples were collected and centrifuged to separate peripheral blood lymphocytes from plasma. Genomic DNA from peripheral blood lymphocytes and cell-free DNA (cfDNA) from plasma were extracted using the RelaxGene Blood DNA System (TianGen Biotech Co., Ltd., Beijing, China) and the QIAamp Circulating Nucleic Acid Kit (Qiagen, Hilden, Germany), respectively. Circulating tumor DNA (ctDNA) was included in cfDNA.

Targeted capture was achieved using a panel of biotinylated DNA probes which cover 605 cancer-related genes (HapOncoCDx™, Roche). Amplified sample libraries and the SeqCap EZ Library were hybridized according to the manufacturer’s protocol. The captured libraries were sequenced using PE150 paired-end sequencing on the NovaSeq 6000 system (Illumina, Inc., San Diego, CA, USA).

### Raw data processing, alignment, mutation calling, and annotation

Raw sequencing data were pre-processed using fastp v0.12.6 (version 0.18.0), which included adapter trimming, removal of low quality bases, and sliding window trimming [15]. Clean reads were aligned to the hg19 genome (GRch37) using Burrows-Wheeler Aligner (version 0.7.15-r1140, default) [16]. Gencore (version 0.12.0) and Samtools (version 0.1.19) were used to remove duplicate reads and to generate pileup files for properly paired reads with mapping quality ≥ 60 [17, 18]. Somatic variants were called using VarScan2 (version 2.3.8) [19]. A manual visual inspection step using GenomeBrowse was applied to remove further artifacts. Somatic mutation calls were annotated using ANNOVAR (version 2018–04-16) [20]. CNVkit (version 0.9.3) and GeneFuse (version 0.6.1) were used for detection of copy number variation and structural variation, respectively [21, 22].

### Statistical analysis

We tested no formal hypotheses in this study. For descriptive analysis of clinical data, categorical data were summarized as counts with percentages and continuous data were summarized as medians with ranges. PFS was depicted using Kaplan–Meier curves and were compared between groups using the log-rank test. Statistical analyses were conducted using SAS 9.4 (SAS Institute, Inc., Cary, North Carolina, USA).

Biomarker analyses were performed in R v2.0.0 and maftools v2.8.0. Fisher’s exact test was used to compare ctDNA mutations between groups. Correlation between *NRAS*-variant allele frequency and tumor evaluations was analyzed using the Pearson correlation coefficient or nonparametric Spearman correlation test and was plotted using the R package ggplot2 (v3.3.5).

All tests were two-sided. Adjusted *P*-values of < 0.05 by Fisher’s exact test were considered statistically significant.

### Data availability

The data generated in this study are available within the article and its appendix files.

## Results

### Patients

Between September 13, 2017, and January 18, 2021, 42 patients were enrolled (median age: 56 years; 47.6% women), including 30 patients in the dose-escalation phase and 12 patients in the dose-expansion phase. Twenty-23 patients (23/42, 54.8%) were acral melanoma, and 13 patients (13/42, 31.0%) were mucosal melanoma. Prior treatment included surgery in 41 patients (41/42, 97.6%), radiotherapy in 5 patients (5/42, 11.9%) and medication in 36 patients (36/42, 85.7%). A total of 17 (17/42, 40.5%) patients had prior treatment with PD-1 and PD-L1 inhibitors. Baseline characteristics are summarized in Table 1.

**Table 1:** Baseline characteristics of included melanoma patients

Characteristic	Total (*N* = 42)
Median age, years (range)	56.0 (31.0, 69.0)
Gender, *N* (%)	
Female	20 (47.6)
Male	22 (52.4)
Median BMI, kg/m^2^ (range)	24.2 (18.4, 36.0)
ECOG performance status, *N* (%)	
0	15 (35.7)
1	27 (64.3)
Pathological type, *N* (%)	
Acral melanoma	23 (54.8)
Mucosal melanoma	13 (31.0)
Other types of melanoma	6 (14.3)
TNM stage, *N* (%)	
III	5 (11.9)
IV	37 (88.1)
Mutant of NRAS, *N* (%)	
Q61R	21 (50.0)
Q61K	7 (16.7)
Q61L	3 (7.1)
Q61H	1 (2.4)
G12D/G12R/G12S/G13C/G60E/G13R	10 (23.8)
Prior treatment, *N* (%)	
Surgery	41 (97.6)
Radiotherapy	5 (11.9)
Medication	36 (85.7)
Prior PD-1/PD-L1 inhibitor, *N* (%)	17 (40.5)

*BMI*, body mass index; *ECOG*, Eastern Cooperative Oncology Group; *PD-1*, programmed cell death 1; *PD-L1* programmed cell death ligand 1

### Pharmacokinetics

HL-085 exposure (area under the curve and maximum plasma concentration) is close to dose proportional over the dose range of 0.5–15 mg twice daily with minimal accumulation. The mean plasma terminal half-life is 21.84 h–34.41 h. More detailed results of the pharmacokinetic analysis of the parent study are described in another article which was recently published by Qian Zhao et al. [23].

### DLT, MTD, and RP2D

In the escalation phase, patients were enrolled into 10 cohorts (0.5–18 mg BID), no DLT were observed, and MTD was not reached. Nine milligrams and above dose can achieve the target exposure, the 15 mg and above dose group had increased safety risks, with drug-related AE leading to discontinuation (cardiotoxicity, ocular toxicity, and interstitial pneumonia), whereas the 12 mg dose group had no drug-related AE leading to discontinuation. As the dose of HL-085 increased, clinical efficacy began to be observed. No subjects achieved PR in the 6 mg dose and below group, while patients achieved PR in the 9 mg and above dose group. The confirmed ORR was 14.3% in all dose groups which was as high as 26.7% in the 12 mg dose group. Based on the available safety, efficacy, pharmacokinetic analysis, and pharmacodynamic analysis, 12 mg BID was identified as the RP2D and selected for dose expansion.

### Safety and tolerability

Safety data for all patients in the study were combined. Table 2 shows drug-related AEs with a frequency of at least 10%. Adverse events in the DLT evaluation period and the whole study period were presented in detail in Additional file 1: Table S1. Drug-related AEs were reported in 36 patients (36/42, 85.7%). The most common AEs in all cohorts were rash (26/42, 61.9%), increased creatine phosphokinase (CK) (25/42, 59.5%), face edema (21/42, 50.0%), increased AST (20/42, 47.6%), peripheral edema (17/42, 40.5%), diarrhea (14/42, 33.3%), increased ALT (14/42, 33.3%), and paronychia (8/42, 19.0%), most of which were grades 1–2. The most frequent grade ≥ 3 AEs were increased CK (3/42, 7.1%), asthenia (3/42, 7.1%), acneiform dermatitis (2/42, 4.8%), and peripheral edema (2/42, 4.8%).

**Table 2:** Drug-related adverse events reported in ≥ 10% of patients during the treatment period

	Total		Grade 1		Grade 2		Grade 3		Grade 4	
Preferred term	12 mg twice daily (*n* = 15)	All doses (*n* = 42)	12 mg twice daily (*n* = 15)	All doses (*n* = 42)	12 mg twice daily (*n* = 15)	All doses (*n* = 42)	12 mg twice daily (*n* = 15)	All doses (*n* = 42)	12 mg twice daily (*n* = 15)	All doses (*n* = 42)
Adverse events related to study drug	15 (100%)	36 (85.7%)	0	6 (14.3%)	6 (40.0%)	14 (33.3%)	7 (46.7%)	13 (31.0%)	2 (13.3%)	3 (7.1%)
Rash	7 (46.7%)	26 (61.9%)	6 (40.0%)	21 (50.0%)	1 (6.7%)	5 (11.9%)	0	0	0	0
Increased creatine phosphokinase	13 (86.7%)	25 (59.5%)	3 (20.0%)	6 (14.3%)	6 (40.0%)	13 (31.0%)	2 (13.3%)	3 (7.1%)	2 (13.3%)	3 (7.1%)
Face edema	12 (80.0%)	21 (50.0%)	11 (73.3%)	19 (45.2%)	0	1 (2.4%)	1 (6.7%)	1 (2.4%)	0	0
Increased aspartate aminotransferase	10 (66.7%)	20 (47.6%)	9 (60.0%)	19 (45.2%)	1 (6.7%)	1 (2.4%)	0	0	0	0
Peripheral edema	9 (60.0%)	17 (40.5%)	6 (40.0%)	14 (33.3%)	1 (6.7%)	1 (2.4%)	2 (13.3%)	2 (4.8%)	0	0
Diarrhea	7 (46.7%)	14 (33.3%)	5 (33.3%)	11 (26.2%)	2 (13.3%)	2 (4.8%)	0	1 (2.4%)	0	0
Increased alanine aminotransferase	6 (40.0%)	14 (33.3%)	5 (33.3%)	13 (31.0%)	1 (6.7%)	1 (2.4%)	0	0	0	0
Paronychia	5 (33.3%)	8 (19.0%)	5 (33.3%)	7 (16.7%)	0	1 (2.4%)	0	0	0	0
Asthenia	4 (26.7%)	7 (16.7%)	2 (13.3%)	3 (7.1%)	1 (6.7%)	1 (2.4%)	1 (6.7%)	3 (7.1%)	0	0
Dermatitis acneiform	4 (26.7%)	6 (14.3%)	2 (13.3%)	3 (7.1%)	1 (6.7%)	1 (2.4%)	1 (6.7%)	2 (4.8%)	0	0
Increased creatine phosphokinase MB	3 (20.0%)	6 (14.3%)	3 (20.0%)	4 (9.5%)	0	2 (4.8%)	0	0	0	0
Proteinuria	4 (26.7%)	6 (14.3%)	3 (20.0%)	4 (9.5%)	1 (6.7%)	2 (4.8%)	0	0	0	0
Skin fissures	4 (26.7%)	5 (11.9%)	4 (26.7%)	5 (11.9%)	0	0	0	0	0	0
Pyrexia	4 (26.7%)	5 (11.9%)	2 (13.3%)	3 (7.1%)	1 (6.7%)	1 (2.4%)	1 (6.7%)	1 (2.4%)	0	0
Decreased white blood cell count	2 (13.3%)	5 (11.9%)	1 (6.7%)	3 (7.1%)	1 (6.7%)	2 (4.8%)	0	0	0	0
Decreased neutrophil count	2 (13.3%)	5 (11.9%)	1 (6.7%)	3 (7.1%)	1 (6.7%)	2 (4.8%)	0	0	0	0
Anemia	4 (26.7%)	5 (11.9%)	2 (13.3%)	3 (7.1%)	2 (13.3%)	2 (4.8%)	0	0	0	0

Data are number of patients (%)

Drug-related AEs leading to drug interruption or dose reduction were observed in 19 patients (19/42, 45.2%); all dose occurred, except 0.5 mg, 1 mg, and 2 mg dose group. The most common AE include the following: increased CK (6/42, 14.3%), fatigue (3/42, 7.1%), peripheral edema, acne-like dermatitis, ambiguous state of consciousness 4.8% (2/42). In this trial, ambiguous state of consciousness were observed in 2 patients, 12 mg and 18 mg dose group respectively. The one occurred in 12 mg dose, died 8 days later due to extensive disease progression and abdominal tumor rupture and hemorrhage; the investigators evaluated that the ambiguous state of consciousness was associated with disease progression. The other one occurred in 18 mg dose, symptoms disappeared and no sequelae after 5 days later. All the others occurred in 1 case.

Drug-related AEs leading to drug discontinuation were only observed in 3 patients (3/42, 7.1%) due to ejection fraction decreased (15 mg cohort), interstitial lung disease (18 mg cohort), and retinal artery occlusion (18 mg cohort). According to the study, DLT were evaluate from 7-day run-in period to the end of the first 28-day treatment cycle. However, the 3 AEs were not occurred during the DLT evaluation period, so they were not considered DLT. No drug-related deaths occurred.

Skin toxicity were the most frequent treatment-related AEs in this study (Table 2) especially for rash, but all of them were grade 1 or 2. The second most common AE was increased CK, most of which were asymptomatic and only a small proportion of patients had mild muscle soreness and fatigue. Six patients (6/42, 14.3%) with increased CK were resolved upon interruption or dose reduction of HL-085. Increased AST, increased ALT, diarrhea, face edema, and peripheral edema were manageable with concomitant medications.

Treatment-related ocular AEs were recorded in 8 patients (8/42, 19%), such as eye discomfort, blurred vision, retinal aneurysm, retinal artery occlusion, retinal hemorrhage, chorioretinopathy, macular edema, cataract, and intraocular hypertension. Most cases of ocular AEs were grade 1–2 and did not require drug suspension or dose reduction; only one patient (retinal artery occlusion, 18 mg cohort) required discontinuation of HL-085 and received concomitant medications (such as Compound Tropicamide Eye Drops, Timolol Maleate Eye Drops, Triamcinolone acetonide acetate injection, compound betamethasone injection).

Most of heart toxicity AEs were grade 1–2 and asymptomatic such as electrocardiogram ST-T segment abnormality, electrocardiogram T wave abnormality, and arrhythmia (grade 1, symptomatic resolution without sequelae). Only one patient with decreased ejection fraction (15 mg cohort) required discontinuation of HL-085.

Respiratory, thoracic, and mediastinal disorders include pneumonitis, dyspnea, interstitial lung disease, and productive cough (each event only in one patient), most of which were grade 1–2. Grade 3 interstitial lung disease (18 mg cohort) was resolved upon discontinuation of HL-085.

### Treatment efficacy

At the time of analysis, among all patients, no patient achieved CR; 6 patients (6/42, 14.3%) achieved confirmed PR; 24 patients (24/42, 57.1%) attained stable disease (SD, Table 3). Clinical efficacy of each dose group were presented in detail in Additional file 1: Table S2. Confirmed objective response rate (ORR) was 14.3% (95% confidence interval [CI]: 5.4%, 28.5%). Disease control rate (DCR) was 78.6%. Median duration of response (DoR) was 3.6 months (95% CI: 0.6, 6.2). Median progression-free survival (PFS) was 3.0 months (95% CI: 2.1, 3.7).

**Table 3:** Tumor response based on investigator assessments

Best overall response	12 mg (*N* = 15)	15 mg (*N* = 3)	18 mg(*N* = 3)	All doses(*N* = 42)
	*n* (%)	*n* (%)	*n* (%)	*n* (%)
CR,	0	0	0	0
PR^#^,	4	0	1	6
SD,	6	3	1	24
ORR^&^ (95%CI)	26.7% (7.8, 55.1)	0 (0.0)	33.3% (0.8, 90.6)	14.3% (5.4, 28.5)
DCR (95%CI)	86.7% (59.5, 98.3)	100.0% (29.2, 100.0)	66.7% (9.4, 99.2)	78.6% (63.2, 89.7)
DOR (months) (95% CI)	2.9 (0.6, 5.5)	NA	/ (/, /)	3.6 (0.6, 6.2)
PFS (months) (95% CI)	3.6 (1.8, 5.5)	4.8 (2.2, 4.8)	7.6 (1.0, 7.6)	3.0 (2.1, 3.7)

^#^Confirmed PR

^&^Confirmed ORR

*CI*, confidence interval; *CR*, complete remission; *DCR*, disease control rate; *DoR*, duration of response; *ORR*, objective response rate; *PFS*, progress-free survival; *PR*, partial remission; *SD*, stable disease; /, not evaluation; *NA*, not available

**Fig. 1:**
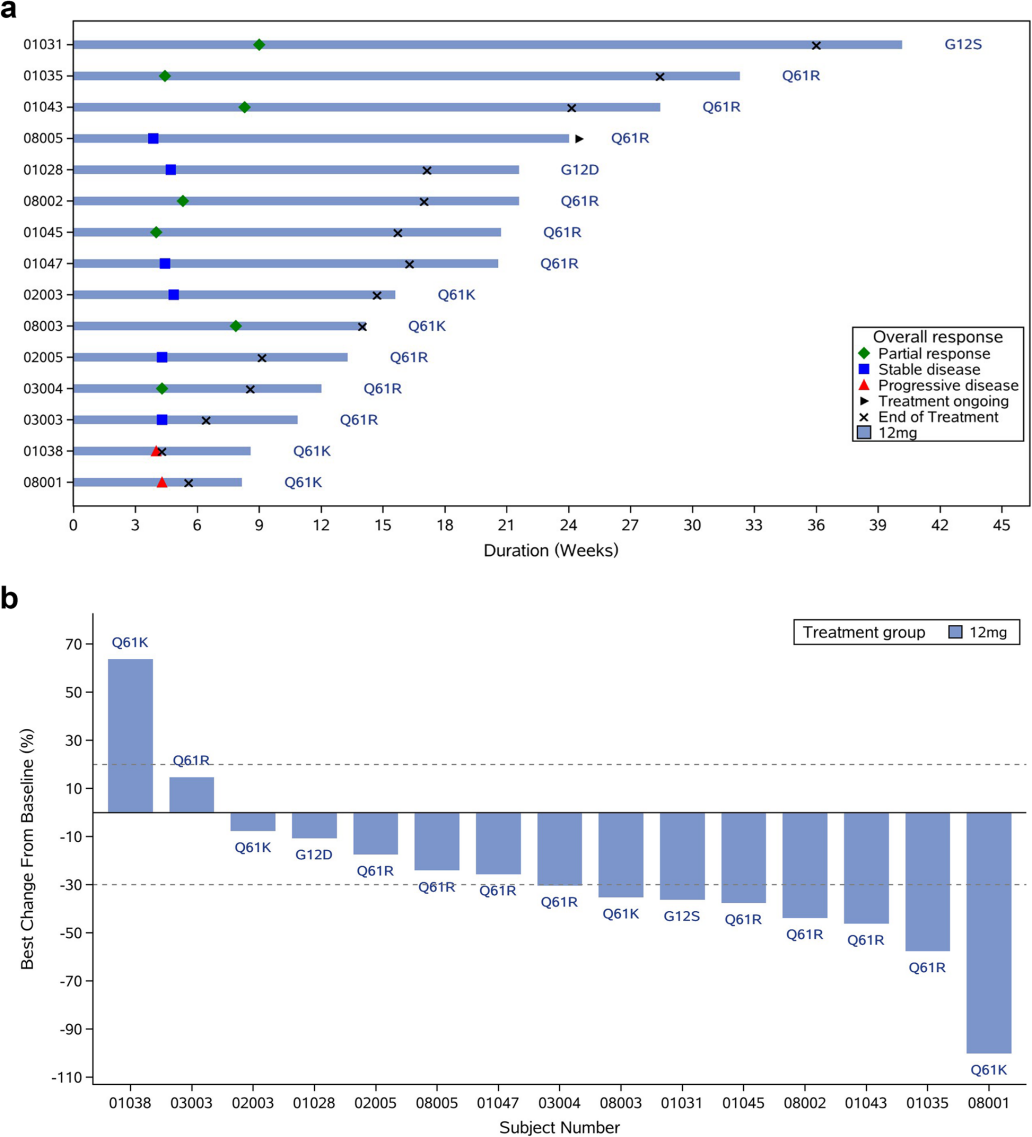
Response to treatment in the 12 mg cohort. Best overall treatment response (**A**) and best tumor change from baseline (**B**)

**Fig. 2:**
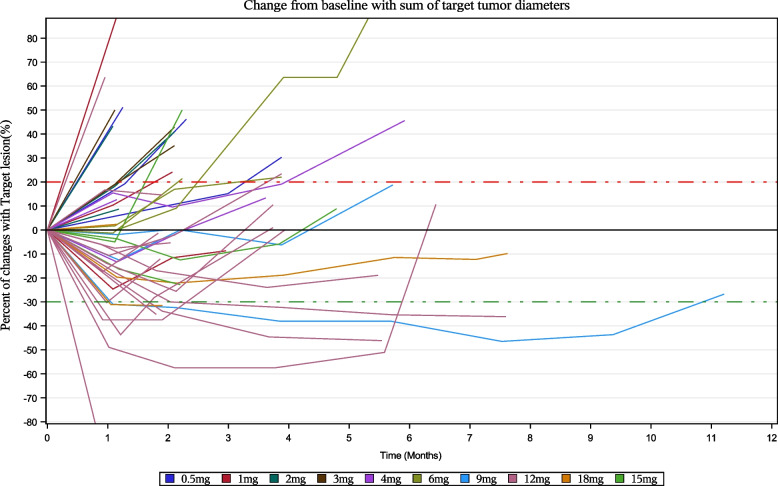
Response depending on the administered dose

In the 12 mg cohort, 4 patients (4/15, 26.7%) achieved confirmed PR and 6 patients (6/15, 40.0%) attained SD, providing confirmed ORR of 26.7% and a DCR of 86.7%. Median DoR in this cohort was 2.9 months (95% CI: 0.6, 5.5), and median PFS was 3.6 months (95% CI: 1.8, 5.5). Best overall response and best tumor change from baseline in the 12 mg cohort during the phase expansion phase is shown in Fig. 1. Spider plot is shown in Fig. 2 to facilitate understanding of the response depending on the administered dose.

In order to evaluate associations between efficacy and pathologic subtypes and *NRAS* mutation types, we performed a subgroup analysis among the 15 patients in the 12 mg cohort who have at least one tumor evaluation. However, pathologic melanoma subtype and NRAS mutation were not significantly correlated with ORR, DCR. The median PFS was 114.0 days [95% CI: 58.0-not evaluation (NE)] in Q61R mutant patients and 64.5 days (28.0-NE) in Q61K mutant patients (*p* = 0.0431). As for pathologic subtypes, there was no statistical difference in the median PFS (*p* = 0.1698). The deficient number of cases may be one reason for this result, whether there is a specific correlation requires further verification by large samples. (Additional file 1: Table S3-S6, Fig. S1(A) and Fig. S1(B)).

### ctDNA analyses

Among 27 patients with baseline blood samples available for ctDNA analysis, *NRAS* mutation was detected in 21 samples (21/27, 78%). Positive *NRAS* mutation status in blood samples was consistent with corresponding tissue samples in 100% of cases (Additional file 1: Fig. S2).

**Fig. 3:**
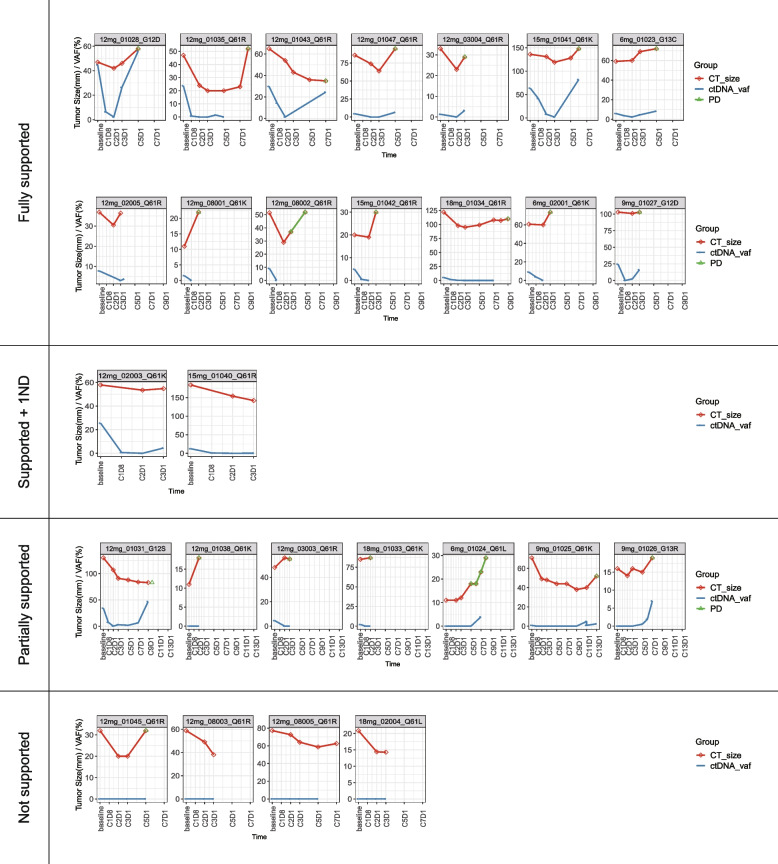
Correlation between *NRAS* variant allele frequency and treatment efficacy. CT, computed tomography. ctDNA, circulating tumor DNA. Vaf, variant allele frequency. PD, progression disease

**Fig. 4:**
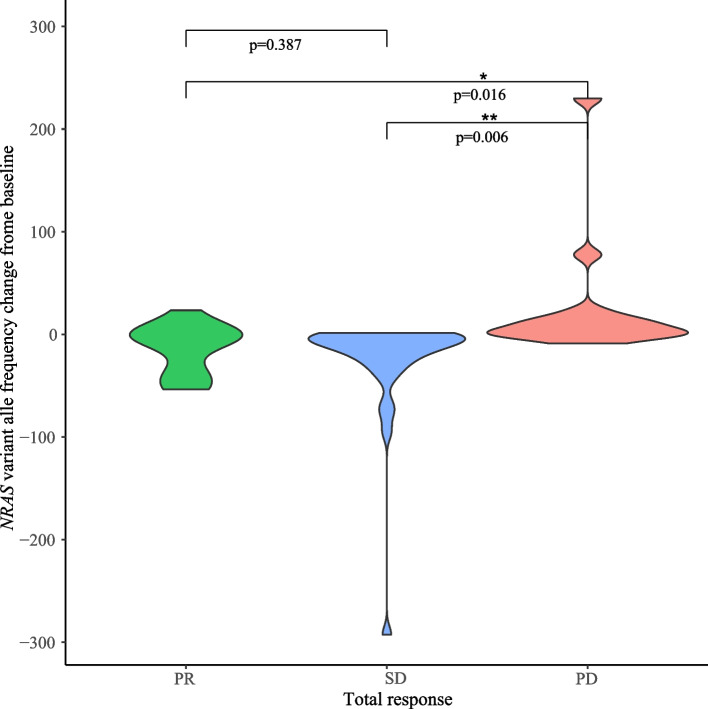
Characterization of *NRAS* variant allele frequency change from baseline in the three groups: partial response (PR), stable disease (SD), and progression disease (PD)

The correlation between *NRAS* variant allele frequency (vaf) and treatment efficacy is presented in Fig. 3. The *NRAS*-vaf change curve completely or partly reflected conventional tumor evaluations in all but 4 patients (in whom *NRAS* mutation was not detectable at all assessments). In the remaining 23 patients, the *NRAS*-vaf change curve completely reflected tumor evaluations in more than half of the patients, while only one or two points failed to reflect tumor evaluation among the other patients (Fig. 3) The *NRAS*-vaf change from baseline in the PD patients was significant different from that in PR patients and SD patients (*p* = 0.016 and *p* = 0.006, respectively; Fig. 4). These data suggest that non-invasive detection of *NRAS*-vaf in ctDNA has high accuracy for predicting disease progression in this setting.

## Discussion

Our findings showed that HL-085 was well tolerated, with the most common AEs of skin-related AEs and increased CK were manageable. No DLT occurred in the dose- escalation phase and MTD was therefore not reached. Anti-tumor activity were first observed in the 4 mg dose cohort with PR first observed at the 9 mg dose, suggesting dose-dependent anti-tumor activity of HL-085. Toxicity of HL-085 increased with dose (respiratory, thoracic and mediastinal disorders, cardiac disorders and eye disorders requiring discontinuation of HL-085 in the 15 and 18 mg dose cohorts). Based on the absence of severe toxicity and promising evidence of efficacy in the dose-escalation phase, 12 mg BID was identified as the RP2D for HL-085 and was selected for dose expansion.

In this study, the most common AEs included rash, increased CK, increased AST, face edema, increase ALT, peripheral edema, and diarrhea. The AEs profile was similar to those seen with other MEK inhibitors in previous trials [13, 24, 25]. The incidence of drug-related grade 3/4 AEs with HL-085 was numerically lower than previously reported for binimetinib, which may be due to differences in study populations (most of melanoma patients in our study were acral melanoma and mucosal melanoma while most patients were cutaneous in Caucasian), or use of a lower dose of HL-085 in this study compared with that of binimetinib in previous trials [26, 27]. Similar to previous studies of MEK inhibitors, ocular toxicity and cardiac toxic effects were also reported in our study but only occurred in one or two patients and most of which were grade 1–2. However, monitoring of ophthalmologic examinations and cardiac function will continue in future studies. The observation of thoracic and mediastinal disorders, cardiac disorders, and eye disorders in the HL-085 15 mg and 18 mg cohorts imply more severe toxicity at higher doses of HL-085, which warrants further attention.

As shown in the spider plot (Fig. 1), during the first imaging assessment period (4 weeks after first administration), target lesion shrinkage was observed in most patients. With the dose of HL-085 increased, clinical efficacy began to be observed, no subjects achieved PR in the 6 mg dose and below group, while patients achieved PR in the 9 mg and above dose group. The efficacy of the RP2D may have an improvement than previous phase 3 of binimetinib in NRAS mutant melanoma (ORR: 26.7% vs 15%, DCR: 86.7% vs 58%, PFS: 3.6 months vs 2.8 months) [13]; we look forward to further validation in a large sample. The promising results with HL-085 might be attributable to the drug’s pharmacokinetic and pharmacodynamics profile, which allows sustained target inhibition and distinguishes HL-085 from other clinically tested MEK inhibitors. Overall, these data indicate the promising potential of HL-085 as a novel therapeutic agent for patients with advanced melanoma with *NRAS* mutations. However, small number of cohort (15 patients) at 12 mg was the limitation and further research was needed in large number cohort.

Syeda et al. [28] conduct a study to explore the association of ctDNA concentrations and efficacy in patients with advanced melanoma treated with dabrafenib or dabrafenib plus trametinib; the results suggests that pretreatment and on-treatment BRAFV600-mutant ctDNA measurements could serve as independent, predictive biomarkers of clinical outcome with targeted therapy. Boerlin et al. [29] reveal that ctDNA was detectable in a statistically significantly larger proportion of patients with distant metastases (79%) than in patients with no distant metastases or only intracranial metastases (32%). Patients with detectable ctDNA was associated with shorter OS in univariate and multivariate analyses. Feng et al. [30] conducted a systematic review and meta-analysis which involved a total of 617 melanoma patients; the results also revealed that compared with baseline undetectable ctDNA patients, detectable ctDNA was highly correlated with poor OS and PFS. Investigation and application of ctDNA will improve “liquid biopsy” and play a role in early prediction, monitoring disease progression, and precise adjusting treatment strategies in melanoma patients. Several previous studies also have indicated that ctDNA could partly reflect treatment efficacy in patients with melanoma [28, 31, 32]. In the present study, *NRAS* mutational status by ctDNA analysis was highly consistent with that observed in tumor tissue. Furthermore, changes in *NRAS* variant allele frequency in ctDNA were highly correlated with changes in tumor size, which is partly in line with a previous study [28]. These findings suggest that ctDNA, as a non-invasive marker, has the potential for predicting disease progression.

## Conclusions

In conclusion, this phase I trial established the RP2D of HL-085 as 12 mg BID in patients with advanced melanoma with *NRAS* mutations. HL-085 had an acceptable tolerability profile and showed promising clinical benefits in this setting, supporting further investigation of HL-085 12 mg BID in clinical trials. Phase 2 monotherapy studies in *NRAS* mutant melanoma and novel combination studies are ongoing.

## Supplementary Information

**Additional file 1.**

## Data Availability

The datasets used and/or analyzed during the current study are available from the corresponding author on reasonable request.

## Abbreviations

AEs: Adverse Events
ALT: Alanine Aminotransferase
AST: Aspartate Aminotransferase
BID: Twice Daily
cfDNA: Cell-free DNA
CI: Confidence Interval
CK: Creatine Phosphokinase
CR: Complete Response
ctDNA: Circulating Tumor DNA
DCR: Disease Control Rate
DLT: Dose-Limiting Toxicity
DoR: Duration of Response
ECOG: Eastern Cooperative Oncology Group
IC50: Half-maximal Inhibitory Concentration
MEK: Mitogen-activated Extracellular Signal-related Kinase
MTD: Maximum Tolerated Dose
ORR: Objective Response Rate
PD: Progressive Disease
PFS: Progression-Free Survival
PR: Partial Response
RECIST: Response Evaluation Criteria in Solid Tumors
RP2D: Recommended Phase II Dose
SD: Stable Disease
vaf: Variant allele frequency

## References

[1] Luke JJ, Flaherty KT, Ribas A, Long GV (2017). Targeted agents and immunotherapies: optimizing outcomes in melanoma. Nat Rev Clin Oncol.

[2] Amin MB, Edge S, Greene F, Byrd DR, Brookland RK, Washington MK (2017). AJCC cancer staging manual.

[3] Balch CM, Gershenwald JE, Soong SJ, Thompson JF, Atkins MB, Byrd DR (2009). Final version of 2009 AJCC melanoma staging and classification. J Clin Oncol.

[4] Naik PP (2021). Cutaneous malignant melanoma: a review of early diagnosis and management. World J Oncol.

[5] Goepfert RP, Myers JN, Gershenwald JE (2020). Updates in the evidence-based management of cutaneous melanoma. Head Neck.

[6] Akbani R, Akdemir KC, Aksoy BA, Albert M, Ally A, Amin SB (2015). Genomic classification of cutaneous melanoma. Cell.

[7] Ottaviano M, Giunta EF, Tortora M, Curvietto M, Attademo L, Bosso D (2021). BRAF gene and melanoma: back to the future. Int J Mol Sci.

[8] Nassar KW, Tan AC (2020). The mutational landscape of mucosal melanoma. Semin Cancer Biol.

[9] Kaufman HL, Margolin K, Sullivan R (2018). Management of metastatic melanoma in 2018. JAMA Oncol.

[10] Jager MJ, Shields CL, Cebulla CM, Abdel-Rahman MH, Grossniklaus HE, Stern MH (2020). Uveal melanoma. Nat Rev Dis Primers.

[11] Indini A, Mandala M (2020). Safety and efficacy evaluation of encorafenib plus binimetinib for the treatment of advanced BRAF-mutant melanoma patients. Expert Opin Drug Saf.

[12] Bertoli E, Giavarra M, Vitale MG, Minisini AM (2019). Neuroblastoma rat sarcoma mutated melanoma: that’s what we got so far. Pigment Cell Melanoma Res.

[13] Dummer R, Schadendorf D, Ascierto PA, Arance A, Dutriaux C, Di Giacomo AM (2017). Binimetinib versus dacarbazine in patients with advanced NRAS-mutant melanoma (NEMO): a multicentre, open-label, randomised, phase 3 trial. Lancet Oncol.

[14] Cheng Y, Tian H (2017). Current development status of MEK inhibitors. Molecules.

[15] Chen S, Zhou Y, Chen Y, Gu J (2018). fastp: an ultra-fast all-in-one FASTQ preprocessor. Bioinformatics.

[16] Li H, Durbin R (2010). Fast and accurate long-read alignment with Burrows-Wheeler transform. Bioinformatics.

[17] Chen S, Zhou Y, Chen Y, Huang T, Liao W, Xu Y (2019). Gencore: an efficient tool to generate consensus reads for error suppressing and duplicate removing of NGS data. BMC Bioinformatics.

[18] Li H, Handsaker B, Wysoker A, Fennell T, Ruan J, Homer N (2009). The sequence alignment/map format and SAMtools. Bioinformatics.

[19] Koboldt DC, Zhang Q, Larson DE, Shen D, McLellan MD, Lin L (2012). VarScan 2: somatic mutation and copy number alteration discovery in cancer by exome sequencing. Genome Res.

[20] Wang K, Li M, Hakonarson H (2010). ANNOVAR: functional annotation of genetic variants from high-throughput sequencing data. Nucleic Acids Res.

[21] Chen S, Liu M, Huang T, Liao W, Xu M, Gu J (2018). GeneFuse: detection and visualization of target gene fusions from DNA sequencing data. Int J Biol Sci.

[22] Talevich E, Shain AH, Botton T, Bastian BC (2016). CNVkit: Genome-wide copy number detection and visualization from targeted DNA sequencing. PLoS Comput Biol.

[23] Zhao Q, Wang T, Wang H, Cui C, Zhong W, Fu D, et al. Phase I pharmacokinetic study of an oral, small-molecule MEK inhibitor tunlametinib in patients with advanced NRAS mutant melanoma. Front Pharmacol. 2022;13:1039416. 10.3389/fphar.2022.1039416 PMC9663925 36386136

[24] Falchook GS, Lewis KD, Infante JR, Gordon MS, Vogelzang NJ, DeMarini DJ (2012). Activity of the oral MEK inhibitor trametinib in patients with advanced melanoma: a phase 1 dose-escalation trial. Lancet Oncol.

[25] Subbiah V, Kreitman RJ, Wainberg ZA, Cho JY, Schellens JHM, Soria JC (2018). Dabrafenib and trametinib treatment in patients with locally advanced or metastatic BRAF V600-mutant anaplastic thyroid cancer. J Clin Oncol.

[26] Bendell JC, Javle M, Bekaii-Saab TS, Finn RS, Wainberg ZA, Laheru DA (2017). A phase 1 dose-escalation and expansion study of binimetinib (MEK162), a potent and selective oral MEK1/2 inhibitor. Br J Cancer.

[27] Watanabe K, Otsu S, Hirashima Y, Morinaga R, Nishikawa K, Hisamatsu Y (2016). A phase I study of binimetinib (MEK162) in Japanese patients with advanced solid tumors. Cancer Chemother Pharmacol.

[28] Syeda MM, Wiggins JM, Corless BC, Long GV, Flaherty KT, Schadendorf D (2021). Circulating tumour DNA in patients with advanced melanoma treated with dabrafenib or dabrafenib plus trametinib: a clinical validation study. Lancet Oncol.

[29] Boerlin A, Bellini E, Turko P, Cheng PF, Levesque MP, Dummer R (2022). The prognostic value of a single, randomly timed circulating tumor DNA measurement in patients with metastatic melanoma. Cancers (Basel).

[30] Feng SN, Cen XT, Tan R, Wei SS, Sun LD (2021). The prognostic value of circulating tumor DNA in patients with melanoma: a systematic review and meta-analysis. Transl Oncol.

[31] Forschner A, Weißgraeber S, Hadaschik D, Schulze M, Kopp M, Kelkenberg S (2020). Circulating Tumor DNA correlates with outcome in metastatic melanoma treated by BRAF and MEK inhibitors - results of a prospective biomarker study. Onco Targets Ther.

[32] Quéreux G, Herbreteau G, Knol AC, Vallée A, Khammari A, Théoleyre S (2017). Efficient treatment of a metastatic melanoma patient with a combination of BRAF and MEK inhibitors based on circulating tumor DNA analysis: a case report. BMC Res Notes.

