# Supplementary material for: First-in-human phase I dose-escalation and dose-expansion trial of the selective MEK inhibitor HL-085 in patients with advanced melanoma harboring NRAS mutations
Source: BMC Med. 2023 Jan 4;21:2. doi: 10.1186/s12916-022-02669-7 (PMC9814429; doi:10.1186/s12916-022-02669-7)
Supplement: Supplementary file 1 — Additional file 1. [file 12916_2022_2669_MOESM1_ESM.docx]

**Additional Table S1.** Adverse events in the DLT evaluation period and the whole study period.

| Items | 0.5 mg | 1 mg | | 2 mg | 3 mg | | 4 mg | | 6 mg | | 9 mg | | 12 mg | | 15 mg | | 18 mg | | Total |
| --- | --- | --- | --- | --- | --- | --- | --- | --- | --- | --- | --- | --- | --- | --- | --- | --- | --- | --- | --- |
|  | N=3 | N=3 | | N=3 | N=3 | | N=3 | | N=3 | | N=3 | | N=15 | | N=3 | | N=3 | | N=42 |
| **DLT evaluation period** | No. (%) | No. (%) | | No. (%) | No. (%) | | No. (%) | | No. (%) | | No. (%) | | No. (%) | | No. (%) | | No. (%) | | No. (%) |
| DLT event | 0 (0.0) | 0 (0.0) | | 0 (0.0) | 0 (0.0) | | 0 (0.0) | | 0 (0.0) | | 0 (0.0) | | 0 (0.0) | | 0 (0.0) | | 0 (0.0) | | 0 (0.0) |
| Total AEs | 3 (100.0) | 3 (100.0) | | 3 (100.0) | 3 (100.0) | | 3 (100.0) | | 3 (100.0) | | 3 (100.0) | | 15 (100.0) | | 3 (100.0) | | 3 (100.0) | | 42 (100.0) |
| Drug-related AEs | 0 (0.0) | 0 (0.0) | | 3 (100.0) | 3 (100.0) | | 3 (100.0) | | 3 (100.0) | | 3 (100.0) | | 15 (100.0) | | 3 (100.0) | | 3 (100.0) | | 36 (85.7) |
| SAEs, No. (%) | 0 (0.0) | 0 (0.0) | | 0 (0.0) | 0 (0.0) | | 0 (0.0) | | 1 (33.3) | | 0 (0.0) | | 1 (6.7) | | 0 (0.0) | | 0 (0.0) | | 2 (4.8) |
| Drug-related SAEs | 0 (0.0) | 0 (0.0) | | 0 (0.0) | 0 (0.0) | | 0 (0.0) | | 1 (33.3) | | 0 (0.0) | | 1 (6.7) | | 0 (0.0) | | 0 (0.0) | | 2 (4.8) |
| Grade ≥ 3 AEs | 0 (0.0) | 0 (0.0) | | 0 (0.0) | 0 (0.0) | | 0 (0.0) | | 1 (33.3) | | 1 (33.3) | | 6 (40.0) | | 1 (33.3) | | 2 (66.7) | | 11 (26.2) |
| Grade ≥ 3 drug-related AEs | 0 (0.0) | 0 (0.0) | | 0 (0.0) | 0 (0.0) | | 0 (0.0) | | 1 (33.3) | | 1 (33.3) | | 6 (40.0) | | 0 (0.0) | | 2 (66.7) | | 10 (23.8) |
| AEs leading to withdrawal | 0 (0.0) | 0 (0.0) | | 0 (0.0) | 0 (0.0) | | 0 (0.0) | | 0 (0.0) | | 0 (0.0) | | 0 (0.0) | | 0 (0.0) | | 0 (0.0) | | 0 (0.0) |
| Drug-related AEs leading to withdrawal | 0 (0.0) | 0 (0.0) | | 0 (0.0) | 0 (0.0) | | 0 (0.0) | | 0 (0.0) | | 0 (0.0) | | 0 (0.0) | | 0 (0.0) | | 0 (0.0) | | 0 (0.0) |
| AEs leading to drug discontinuation | 0 (0.0) | 0 (0.0) | | 0 (0.0) | 0 (0.0) | | 0 (0.0) | | 0 (0.0) | | 0 (0.0) | | 0 (0.0) | | 0 (0.0) | | 0 (0.0) | | 0 (0.0) |
| Drug-related AEs leading to drug discontinuation | 0 (0.0) | 0 (0.0) | | 0 (0.0) | 0 (0.0) | | 0 (0.0) | | 0 (0.0) | | 0 (0.0) | | 0 (0.0) | | 0 (0.0) | | 0 (0.0) | | 0 (0.0) |
| **The whole study period** | | | | | | | | | | | | | | | | | | | |
| Total AEs | 3 (100.0) | 3 (100.0) | | 3 (100.0) | 3 (100.0) | | 3 (100.0) | | 3 (100.0) | | 3 (100.0) | | 15 (100.0) | | 3 (100.0) | | 3 (100.0) | | 42 (100.0) |
| Drug-related AEs | 0 (0.0) | 0 (0.0) | | 3 (100.0) | 3 (100.0) | | 3 (100.0) | | 3 (100.0) | | 3 (100.0) | | 15 (100.0) | | 3 (100.0) | | 3 (100.0) | | 36 (85.7) |
| SAEs, No. (%) | 0 (0.0) | 0 (0.0) | | 0 (0.0) | 0 (0.0) | | 0 (0.0) | | 1 (33.3) | | 0 (0.0) | | 5 (33.3) | | 1 (33.3) | | 2 (66.7) | | 9 (21.4) |
| Drug-related SAEs | 0 (0.0) | 0 (0.0) | | 0 (0.0) | 0 (0.0) | | 0 (0.0) | | 1 (33.3) | | 0 (0.0) | | 2 (13.3) | | 1 (33.3) | | 2 (66.7) | | 6 (14.3) |
| Grade ≥ 3 AEs | 0 (0.0) | 0 (0.0) | | 0 (0.0) | 1 (33.3) | | 2 (66.7) | | 1 (33.3) | | 1 (33.3) | | 10 (66.7) | | 3 (100.0) | | 2 (66.7) | | 20 (47.6) |
| Grade ≥ 3 drug-related AEs | 0 (0.0) | 0 (0.0) | | 0 (0.0) | 0 (0.0) | | 0 (0.0) | | 1 (33.3) | | 1 (33.3) | | 9 (60.0) | | 3 (100.0) | | 2 (66.7) | | 16 (38.1) |
| AEs leading to withdrawal from the trial | 0 (0.0) | 0 (0.0) | | 0 (0.0) | 0 (0.0) | | 0 (0.0) | | 0 (0.0) | | 0 (0.0) | | 2 (13.3) | | 0 (0.0) | | 0 (0.0) | | 2 (4.8) |
| Drug-related AEs leading to withdrawal from the trial | 0 (0.0) | 0 (0.0) | | 0 (0.0) | 0 (0.0) | | 0 (0.0) | | 0 (0.0) | | 0 (0.0) | | 0 (0.0) | | 0 (0.0) | | 0 (0.0) | | 0 (0.0) |
| AEs leading to drug discontinuation | 0 (0.0) | 0 (0.0) | | 0 (0.0) | 0 (0.0) | | 0 (0.0) | | 0 (0.0) | | 0 (0.0) | | 1 (6.7) | | 1 (33.3) | | 2 (66.7) | | 4 (9.5) |
| Drug-related AEs leading to drug discontinuation | 0 (0.0) | 0 (0.0) | | 0 (0.0) | 0 (0.0) | | 0 (0.0) | | 0 (0.0) | | 0 (0.0) | | 0 (0.0) | | 1 (33.3) | | 2 (66.7) | | 3 (7.1) |
| AEs leading to drug interruption or dose reduction | 0 | 0 | 0 | 1 (33.3%) | | 1(33.3%) | | 1(33.3%) | | 1(33.3%) | | 10(66.7%) | | 3(100%) | | 2(66.7%) | | 19(45.2%) | |
| Drug-related AEs leading to drug interruption or dose reduction | 0 | 0 | 0 | 1 (33.3%) | | 1(33.3%) | | 1(33.3%) | | 1(33.3%) | | 10(66.7%) | | 3(100%) | | 2(66.7%) | | 19(45.2%) | |

DLT, dose limited toxicity; AEs, adverse events; SAEs, severe adverse events.

**Additional Table S2.** Clinical efficacy in the whole study period.

| Items | 0.5 mg | 1 mg | 2 mg | 3 mg | 4 mg | 6 mg | 9 mg | 12 mg | 15 mg | 18 mg | Total |
| --- | --- | --- | --- | --- | --- | --- | --- | --- | --- | --- | --- |
|  | N=3 | N=3 | N=3 | N=3 | N=3 | N=3 | N=3 | N=15 | N=3 | N=3 | N=42 |
| CR, No. (%) | 0 (0.0) | 0 (0.0) | 0 (0.0) | 0 (0.0) | 0 (0.0) | 0 (0.0) | 0 (0.0) | 0 (0.0) | 0 (0.0) | 0 (0.0) | 0 (0.0) |
| PR, No. (%) | 0 (0.0) | 0 (0.0) | 0 (0.0) | 0 (0.0) | 0 (0.0) | 0 (0.0) | 1 (33.3) | 4 (26.7) | 0 (0.0) | 1 (33.3) | 6 (14.3) |
| SD, No. (%) | 1 (33.3) | 1 (33.3) | 0 (0.0) | 0 (0.0) | 2 (66.7) | 2 (66.7) | 1 (33.3) | 6 (40.0) | 2 (66.7) | 1 (33.3) | 16 (38.1) |
| ORR, No. (%) | 0 (0.0) | 0 (0.0) | 0 (0.0) | 0 (0.0) | 0 (0.0) | 0 (0.0) | 1 (33.3) | 4 (26.7) | 0 (0.0) | 1 (33.3) | 6 (14.3) |
| 95%CI | /, / | /, / | /, / | /, / | /, / | /, / | 0.8, 90.6 | 7.8, 55.1 | /, / | 0.8, 90.6 | 5.4, 28.5 |
| DCR, No. (%) | 1 (33.3) | 1 (33.3) | 0 (0.0) | 0 (0.0) | 2 (66.7) | 2 (66.7) | 2 (66.7) | 10 (66.7) | 2 (66.7) | 2 (66.7) | 22 (52.4) |
| 95%CI | 0.8, 90.6 | 0.8, 90.6 | /, / | /, / | 9.4, 99.2 | 9.4, 99.2 | 9.4, 99.2 | 38.4, 88.2 | 9.4, 99.2 | 9.4, 99.2 | 36.4, 68.0 |
| DOR (months) |  |  |  |  |  |  |  |  |  |  |  |
| 25th percentile | / | / | / | / | / | / | 10.1 | 0.9 | / | / | 1.5 |
| 95%CI | /, / | /, / | /, / | /, / | /, / | /, / | /, / | 0.6, 2.9 | /, / | /, / | 0.6, 3.6 |
| median | / | / | / | / | / | / | 10.1 | 2.9 | / | / | 3.6 |
| 95%CI | /, / | /, / | /, / | /, / | /, / | /, / | /, / | 0.6, 5.5 | /, / | /, / | 0.6, 6.2 |
| 75th percentile | / | / | / | / | / | / | 10.1 | 5.5 | / | / | 6.2 |
| 95%CI | /, / | /, / | /, / | /, / | /, / | /, / | /, / | 1.5, 6.2 | /, / | /, / | 1.5, 10.1 |
| Range | /, / | /, / | /, / | /, / | /, / | /, / | 10.1 10.1 | 0.6,6.2 | /, / | 0.9, 0.9 | 0.6, 10.1 |
| 3-month DOR rate (%) | / | / | / | / | / | / | 100.0 | 42.9 | / | / | 50.8 |
| 95%CI | /, / | /, / | /, / | /, / | /, / | /, / | /, / | 9.8, 73.4 | /, / | /, / | 15.7, 78.1 |
| 6-month DOR rate (%) | / | / | / | / | / | / | 100.0 | 14.3 | / | / | 25.4 |
| 95%CI | /, / | /, / | /, / | /, / | /, / | /, / | /, / | 0.7, 46.5 | /, / | /, / | 3.8, 56.4 |
| 12-month DOR rate (%) | / | / | / | / | / | / | 0.0 | 0.0 | / | / | 0.0 |
| 95%CI | /, / | /, / | /, / | /, / | /, / | /, / | /, / | /, / | /, / | /, / | /, / |
| PFS (months) |  |  |  |  |  |  |  |  |  |  |  |
| 25th percentile | 1.2 | 1.2 | 1.1 | 1.1 | 1.1 | 2.2 | 2.0 | 1.9 | 2.2 | 1.0 | 1.9 |
| 95%CI | 1.2, 3.9 | 1.2, 3.0 | 1.1, 2.1 | 1.1, 2.1 | 1.1, 5.9 | 2.2, 3.9 | 2.0, 11.2 | 0.9, 3.5 | 2.2, 4.8 | 1.0, 7.6 | 1.1, 2.1 |
| median | 2.3 | 2.1 | 1.1 | 2.1 | 3.4 | 3.9 | 5.7 | 3.6 | 4.8 | 7.6 | 3.0 |
| 95%CI | 1.2, 3.9 | 1.2, 3.0 | 1.1, 2.1 | 1.1, 2.1 | 1.1, 5.9 | 2.2, 3.9 | 2.0, 11.2 | 1.8, 5.5 | 2.2, 4.8 | 1.0, 7.6 | 2.1, 3.7 |
| 75th percentile | 3.9 | 3.0 | 2.1 | 2.1 | 5.9 | 3.9 | 11.2 | 5.5 | 4.8 | 7.6 | 3.9 |
| 95%CI | 1.2, 3.9 | 1.2, 3.0 | 1.1, 2.1 | 1.1, 2.1 | 1.1, 5.9 | 2.2, 3.9 | 2.0, 11.2 | 3.5, 8.3 | 2.2, 4.8 | 1.0, 7.6 | 3.5, 6.4 |
| Range | 1.2, 3.9 | 1.2, 3.0 | 1.1, 2.1 | 1.1, 2.1 | 1.1, 5.9 | 2.2, 3.9 | 2.0, 11.2 | 0.9, 8.3 | 2.2, 4.8 | 1.0, 7.6 | 0.9, 11.2 |
| 3-month PFS (%) | 33.3 | 0.0 | 0.0 | 0.0 | 66.7 | 66.7 | 66.7 | 65.5 | 66.7 | 66.7 | 48.1 |
| 95%CI | 0.9, 77.4 | /, / | /, / | /, / | 5.4, 94.5 | 5.4, 94.5 | 5.4, 94.5 | 35.7, 84.0 | 5.4, 94.5 | 5.4, 94.5 | 32.1, 62.5 |
| 6-month PFS (%) | 0.0 | 0.0 | 0.0 | 0.0 | 0.0 | 0.0 | 33.3 | 21.8 | 0.0 | 66.7 | 12.5 |
| 95%CI | /, / | /, / | /, / | /, / | /, / | /, / | 0.9, 77.4 | 5.3, 45.4 | /, / | 5.4, 94.5 | 4.2, 25.4 |
| 12-month PFS (%) | 0.0 | 0.0 | 0.0 | 0.0 | 0.0 | 0.0 | 0.0 | 0.0 | 0.0 | 0.0 | 0.0 |
| 95%CI | /, / | /, / | /, / | /, / | /, / | /, / | /, / | /, / | /, / | /, / | /, / |

CI, confidence interval; CR, complete remission; DCR, disease control rate; DoR, duration of response; ORR, objective response rate; PFS, progress-free survival; PR, partial remission; SD, stable disease; /, not evaluated.

**Additional Table S3.** Correlation between tumor pathology type and ORR.

| Tumor site | Confirmed ORR | |  |
| --- | --- | --- | --- |
|  | No | Yes | Total |
| Acral melanoma, No. (%) | 4 (57.1) | 3 (42.9) | 7 |
| Mucosal melanoma, No. (%) | 2 (100.0) | 0 (0.0) | 2 |
| Other, No. (%) | 2 (66.7) | 1 (33.3) | 3 |
| Total | 8 | 4 | 12 |
| *P* value | 0.745 | |  |

DCR, disease control rate; ORR, objective response rate.

**Additional Table S4.** Correlation between tumor pathology type and DCR.

| Tumor site | DCR | |  |
| --- | --- | --- | --- |
|  | No | Yes | Total |
| Acral melanoma, No. (%) | 2 (28.6) | 5 (71.4) | 7 |
| Mucosal melanoma, No. (%) | 0 (0.0) | 2 (100.0) | 2 |
| Other, No. (%) | 0 (0.0) | 3 (100.0) | 3 |
| Total | 2 | 10 | 12 |
| *P* value |  | 1.000 |  |

DCR, disease control rate; ORR, objective response rate.

**Additional Table S5.** Correlation between Q61R/Q61K mutation and ORR.

| NRAS mutation site | Confirmed ORR | |  |
| --- | --- | --- | --- |
|  | No | Yes | Total |
| Other, No. (%) | 1 (50.0) | 1 (50.0) | 2 |
| Q61K, No. (%) | 2 (100.0) | 0 (0.0) | 2 |
| Q61R, No. (%) | 5 (62.5) | 3 (37.5) | 8 |
| Total | 8 | 4 | 12 |
| *P* value | 1.000 | |  |

DCR, disease control rate; ORR, objective response rate.

**Additional Table S6.** Correlation between Q61R/Q61K mutation and DCR.

| NRAS mutation site | DCR | |  |
| --- | --- | --- | --- |
|  | No | Yes | Total |
| Other, No. (%) | 0 (0.0) | 2 (100.0) | 2 |
| Q61K, No. (%) | 0 (0.0) | 2 (100.0) | 2 |
| Q61R, No. (%) | 2 (25.0) | 6 (75.0) | 8 |
| Total | 2 | 10 | 12 |
| *P* value |  | 1.000 |  |

DCR, disease control rate; ORR, objective response rate.


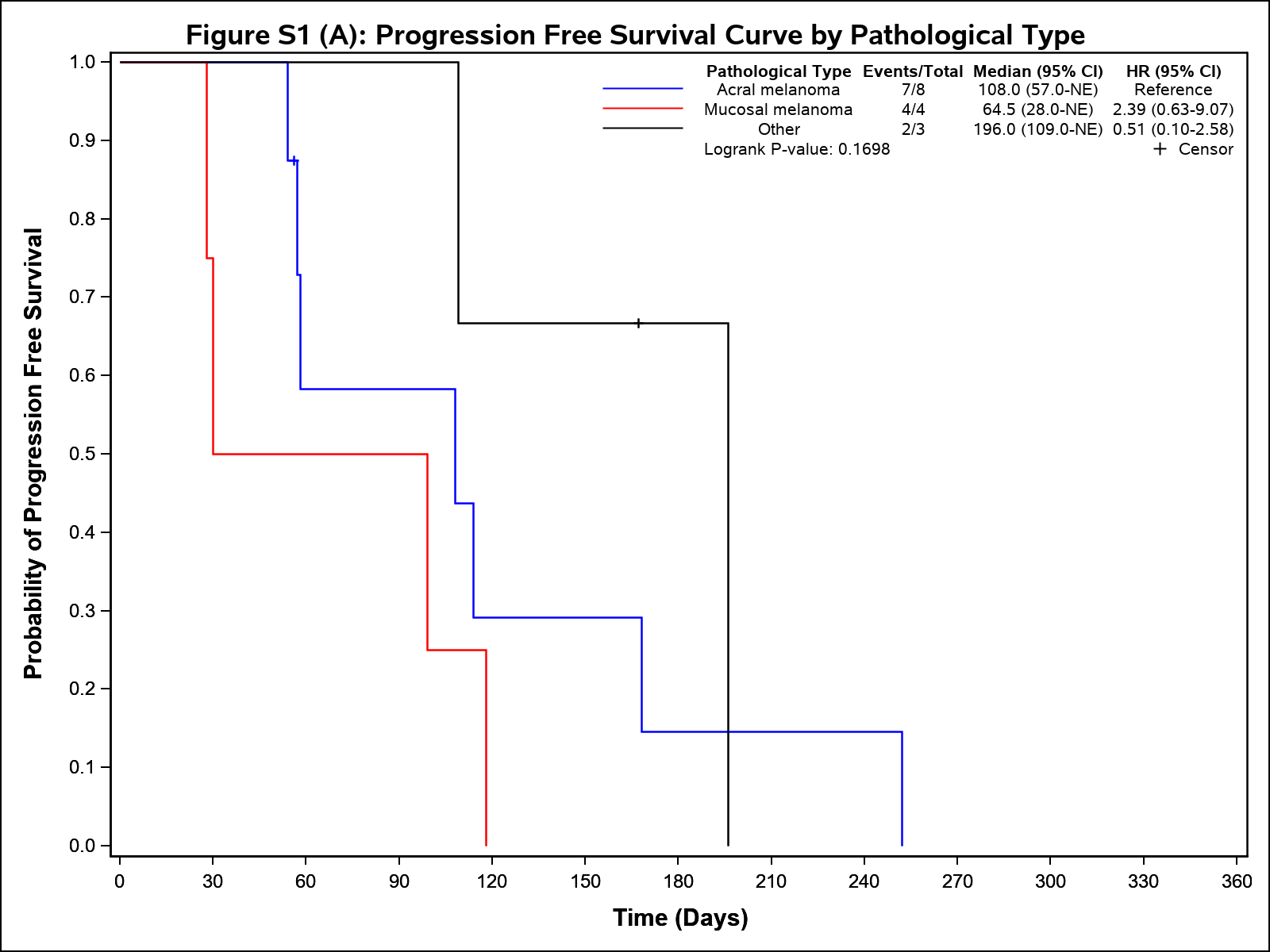


**Additional** **Figure S1** (A). Correlation between progression-free survival and pathologic melanoma subtype.


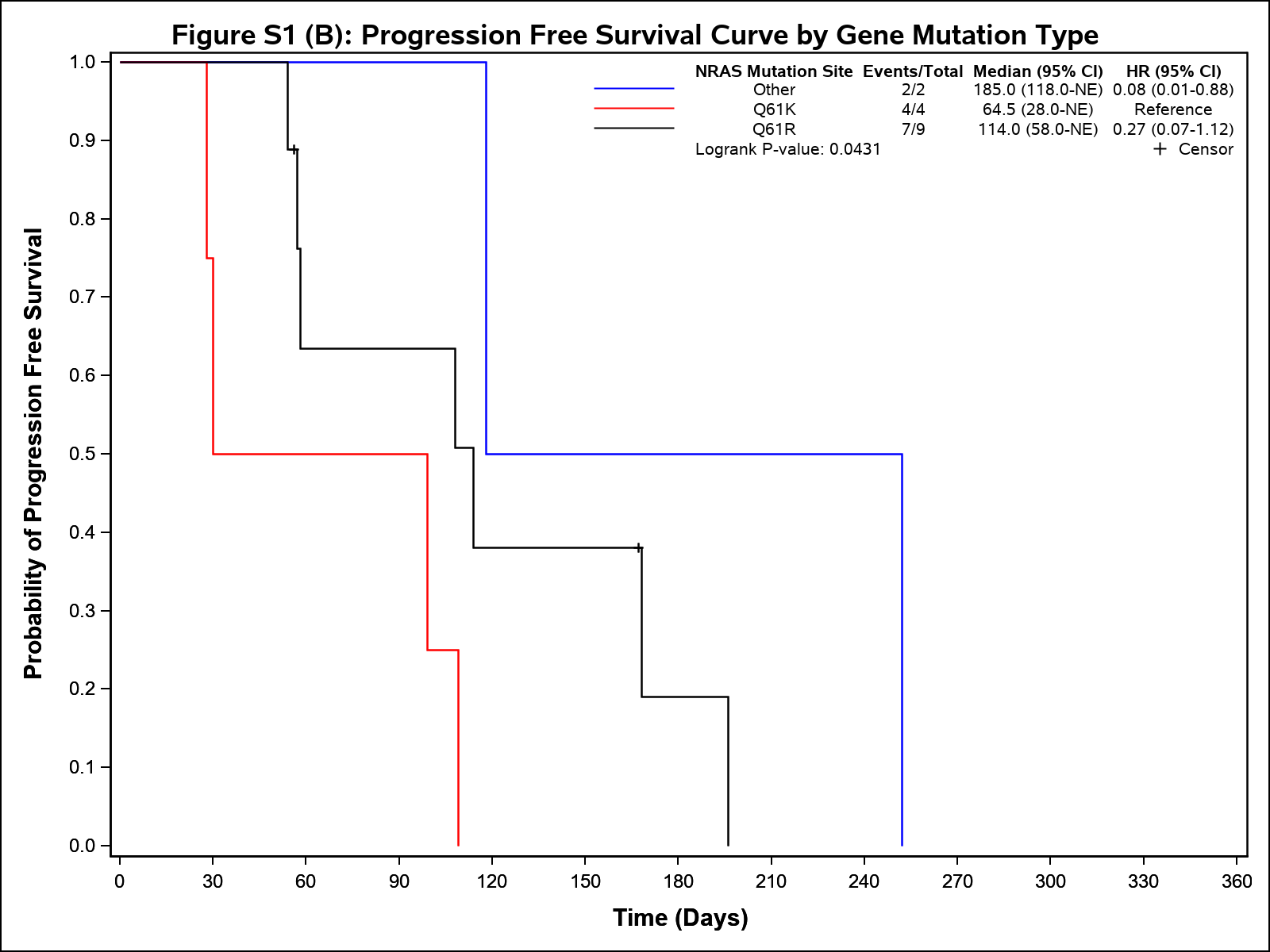


**Additional Figure S1** (B). Correlation between progression-free survival and *NRAS* mutation type.


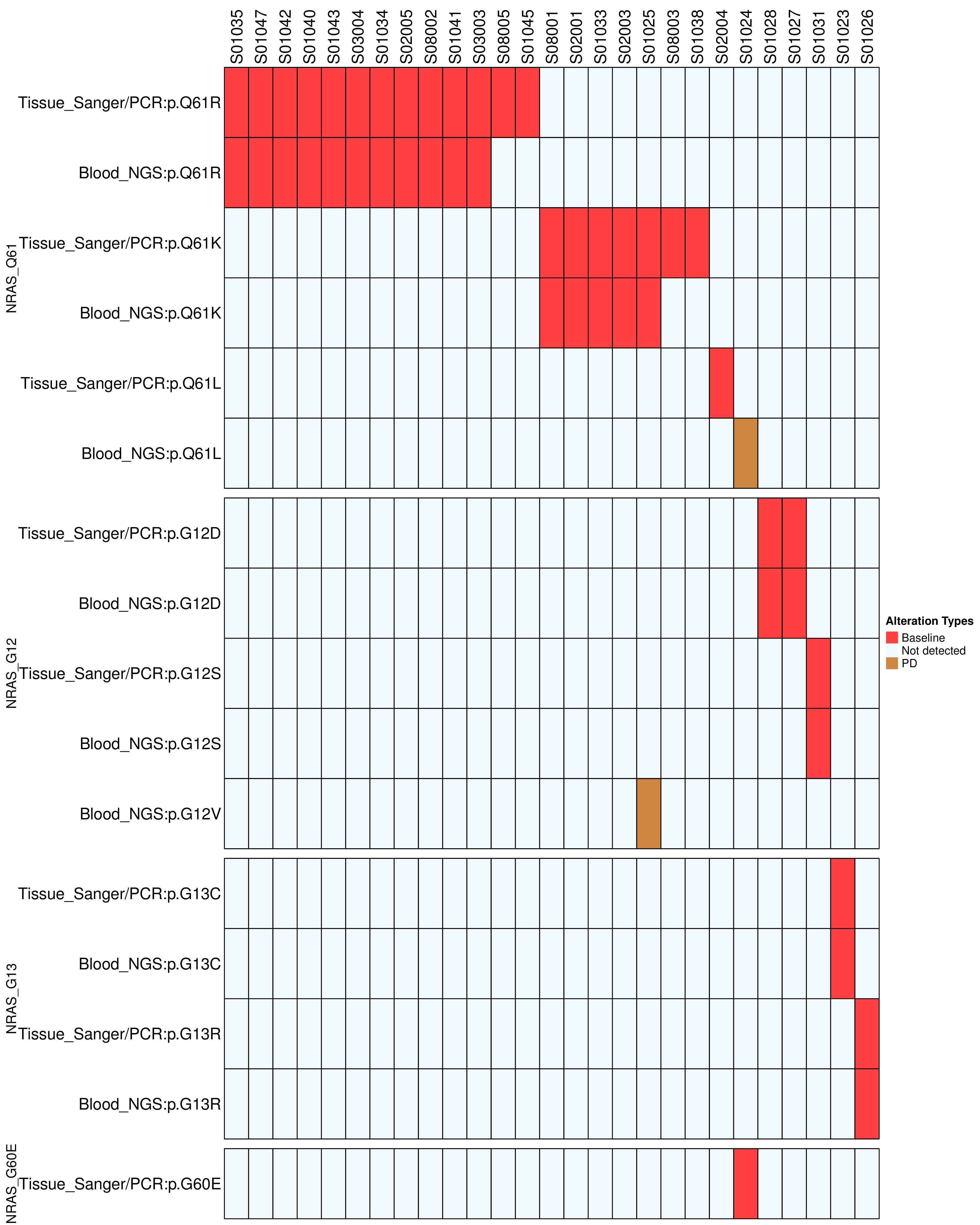


**Additional Figure S2.** NRAS mutation analysis in blood samples and tissue samples.
